# Influence of physical activity and postural habits in schoolchildren with scoliosis

**DOI:** 10.1186/s13690-021-00584-6

**Published:** 2021-04-29

**Authors:** Sanderson José Costa de Assis, Geronimo José Bouzas Sanchis, Clécio Gabriel de Souza, Angelo Giuseppe Roncalli

**Affiliations:** 1 Postgraduate program in public health at the Federal University of Rio Grande do Norte, Natal, Rio Grande do Norte Brazil; 2 Postgraduate program in rehabilitation sciences at the Federal University of Rio Grande do Norte, Santa Cruz, Rio Grande do Norte Brazil

**Keywords:** Posture, Exercise, Risk factors

## Abstract

**Background:**

Scoliosis is considered one of the main musculoskeletal changes in childhood, and is characterized by three-dimensional changes in the spine. Schoolchildren is a group who are directly exposed to this condition because they go through a rapid growth phase in adolescence, added to other external factors such as school environment and daily living habits such as little physical activity. This study aimed to identify the risk factors associated with scoliosis in schoolchildren.

**Methods:**

An observational, retrospective case control study with a quantitative approach was carried out in the city of Santa Cruz/RN. The presence of scoliosis was assessed using the Adams test and physical activity by the Daily Physical Activity Index (IPAQ) and by a questionnaire on competitive sports practice, in addition to a questionnaire on postural habits in childhood and adolescence. Conditional multiple logistic regression was performed for statistical analysis, and the adjusted Odds Ratios (OR) and the respective confidence intervals (95%) of the outcome variable were estimated.

**Results:**

A total of 156 schoolchildren participated in the study, with an average age of 13.9 years, with 55.1% being female and 44.9% male, attending between the 6th grade of elementary school and the 3rd year of high school. Furthermore, 42.9% of these participants were considered irregularly active and only 33.3% practiced physical activity on a regular basis. After bivariate analysis and conditional logistic regression, little physical activity was shown to be a risk factor for scoliosis (*p* = 0.041; OR: 2.81; 95% CI: 1.04–7.57), while the postural habits evaluated in this study did not show a statistical association with scoliosis.

**Conclusion:**

Low practice of physical activity and schoolchildren being classified as irregularly active were considered as risk factors for scoliosis, however postural habits do not seem to be associated with this condition.

## Key points

- No association was observed between postural habits and scoliosis in schoolchildren.
- Physical activity appears to be a protective factor for scoliosis in schoolchildren.
- It is necessary to rethink professional practice for the prevention of scoliosis.

## Background

Body posture is defined as the ideal position adopted by human beings for their daily activities through their body structures and function in order to have better biomechanical efficiency with less energy expenditure. However, this is not always possible, as habits are often adopted which may disregard this pattern and lead to postural changes [1, 2].

Schoolchildren are one of the main affected groups by scoliosis due to their environment which causes them to adopt inappropriate postures from school furniture, long permanence of sitting posture, excess weight and asymmetrical backpack support, and from their daily life habits adopted within and out of that environment [3–6].

Regarding their daily habits, there is an increasing use of cell phones, video games and desktop computers which can increasingly encourage sedentary behavior in childhood [7, 8]. Sedentary behavior is associated with several diseases, including cardiovascular disorders, hypertension, diabetes and musculoskeletal disorders. In turn, the latter may be associated with undesirable postures in students in addition to causing discomfort [9–11].

The period of rapid growth in adolescents, also called the “growth spurt phase”, contributes to the presence of postural changes occurring in childhood and adolescence, especially scoliosis [7, 8]. The prevalence of scoliosis in schoolchildren varies around 22% in the Brazilian population and there is a tendency for the postural problem to worsen in adolescence and adulthood [12–15]. As a consequence, it can result in spinal diseases and future low back pain, which are today configured as a public health problem with increasing treatment costs, as well as long-term social security expenses in view of the high amount of absenteeism and retirement due to such factors, in addition to affecting the quality of life of individuals [12–16].

When considering that epidemiological studies are the first steps for elaborating health promotion and disease prevention programs, including postural assessment practices in schools for the early identification of kinetic-functional postural changes is justified, as well as the fact that schoolchildren constitute a population with risk potential. Thus, this study aimed to identify the risk factors associated with scoliosis in schoolchildren from a 5-year retrospective analysis.

## Materials and methods

### Study design

This is an observational case-control study based on a retrospective analysis of a period of 5 years. It was held in the city of Santa Cruz, Rio Grande do Norte, Brazil. It was approved by the Research Ethics Committee of the Onofre Lopes University Hospital of the Federal University of Rio Grande do Norte - CEP/HUOL, under opinion No. 1,327,521.

### Study population

The study population consisted of students aged between 12 and 17 years old from public schools in the municipality of Santa Cruz/RN. In order to obtain the sample, schoolchildren who participated in a previous cross-sectional study [17] in the same municipality in 2011, and had a positive Adams sign for scoliosis were considered cases. In order to find the participants again, a screening was carried out and new confirmation tests for scoliosis were performed. Participants considered to be controls in this study were selected from the pairing with schoolchildren who had some common characteristics such as gender, age and height, but without a diagnosis of scoliosis.

The inclusion criteria for the study were children and adolescents aged between 12 and 17 years old enrolled in the public education network of the municipality of Santa Cruz/RN and obtaining a signature from their parent or guardian on the Free and Informed Consent Form, as well as an assent form by the schoolchild.

The study excluded individuals who underwent any corrective spinal surgical procedure before the data collection process, children and adolescents with physical and and/or mental disabilities and orthopedic, traumatological and/or rheumatological injuries which prevented the maintenance of the position orthostatic and those who refused to participate in the study again.

### Instruments used

The Adams test was used to assess scoliosis, which is a clinical test that evaluates the rotational component of scoliosis and is considered the main screening test for scoliosis due to its practicality and low cost. It is considered a test with good accuracy compared to the analysis by the Cobb angle, with sensitivity values ranging from 74 to 100% and specificity from 60 to 99%, varying according to the location and magnitude of the curve [18, 19].

Life habits were assessed based on the variables of physical activity, competitive sports and postural habits. The physical activity level was assessed as an indicator of active or sedentary lifestyle habits using the International Physical Activity Questionnaire (IPAQ) short version, translated and validated for Brazil [20, 21].

For the IPAQ classification, sedentary individuals were those who did not perform physical activity for at least 10 continuous minutes for 5 years. The irregularly active ones were classified as type A and B. The irregular ones for type A physical activity were those who performed activities for up to 5 days a week or lasting 150 min/week. The irregularly active-B were those who did not meet the criteria of recommendation A as to frequency and duration. Active people are those who performed vigorous activities, at least 3 days a week and lasting 20 min or more per session. The activities carried out in the last 5 years were taken into account due to the retrospective time adopted in this study.

In order to evaluate competitive sports practice, the students were asked about the activity performance, with competitive sports practice being considered as any sport practiced with the intention of competing with other people in tournaments, championships or sporting events [22]. Then they were asked about the sport they were playing, for how long they played and the period and frequency of competitive sports.

For the assessment of posture-related lifestyle habits, the activities that students spent most of their time from childhood to adolescence were evaluated based on the Rebolho questionnaire [23] and adapted for this study, including: sitting position, transportation of school supplies, gesture to pick up an object on the floor and sleeping habits.

A recall for the analysis of these postural habits was performed about the postural habits adopted in the last 5 years. The student visualized figures with different postures, and then they chose the option that best suited their reality, without necessarily being the one they considered correct [23].

In order to identify the association between scoliosis and the study’s independent variables, postural habits were dichotomized into adequate and inadequate; physical activity was categorized into active, irregularly active and sedentary; and scoliosis was defined as absent or present.

### Examiners

The examiners in this study were previously trained for a period of 2 months and a Kappa index higher than 0.7 was observed among the evaluators, supporting the good reproducibility of the tests for postural evaluation.

### Statistical analysis

The sample calculation was based on data of the proportion between postural habits and scoliosis and further based on data from the study by Baroni et al. (2015) [17], estimating an Odds Ratio of 2.5 and a significance level of 5%, which projected a total of 156 participants, distributed in 78 cases and 78 for the control considering a ratio of one control to each case.

Demographic characteristics, gender, age, physical activity, competitive sports practice and postural habits were described using simple tables. Initially a bivariate analysis was performed between the outcome variable and the independent variables. Variables with *p*-value less than 0.25 and which fit the theoretical model were included in the multiple analysis. For being a study with a dichotomous outcome variable and for having several explanatory variables for the outcome, in addition to being a matched sample, conditional logistic regression was used in the inferential statistical analysis, and the Odds ratio (OR) and the respective confidence intervals (95%) of the outcome variable were estimated and a significance level of 5% (α < 0.05) was adopted.

## Results

### General characteristics of study population

A total of 156 participants participated in the study (78 belonging to the cases and 78 considered as controls), aged between 12 and 17 years old, students from the 6th grade of elementary school to the 3rd grade of high school, enrolled in the public school system in the municipality of Santa Cruz/RN.

A total of 55.1% (86) of the students were female and the average age of the students was 13.9 years. Regarding postural habits, only 19.9% adopted a posture considered adequate for sitting with most of their lower back against the chair; 48.7% sat without clenching the glutes and 41.7% supported their feet on the floor in an appropriate manner. Backpack transport was carried out correctly by 71.2% of the students, while only 30.1% picked up objects properly from the floor. The main way to sleep was 64.7% in a bed; 6.4% in a hammock; and 28.8% in both (Table 1).

**Table 1:** General characteristics of study population. Santa Cruz, RN

Variable	n (%)
**Gender**	
Male	70 (44.9)
Female	86 (55.1)
**Physical activity**	
Active	52 (33.3)
Irregularly active	67 (42.9)
Sedentaryo	37 (23.7)
**Competitive sports practice**	
Yes	34 (21.8)
No	122 (78.2)
**Spinal posture when sitting**	
Adequate	31 (19.9)
Inadequateo	125 (80.1)
**Buttocks positioning while sitting**	
Adequate	76 (48.7)
Inadequate	80 (51.3)
**Feet positioning while sitting**	
Adequate	65 (41.7)
Inadequate	91 (58.3)
**Backpack transport**	
Adequate	111 (71.2)
Inadequate	45 (28.8)
**Posture while picking up an object from the floor**	
Adequate	47 (30.1)
Inadequate	109 (69.9)
**Sleeping**	
Bed	101 (64.7)
Hammock	10 (6.4)
Both	45 (28.8)

Regarding the physical activity level, 33.3% of students were considered active; 42.9% irregularly active; and 23.7% sedentary, with competitive sports being present in 21.8% of students (Table 1).

### Association between “scoliosis” and the selected variables

Physical activity showed statistical significance (*p* = 0.016), showing that those who performed little physical activity (irregularly active) have a higher risk of having scoliosis when compared to active students (OR: 2.81; 95% CI: 1.21–6.49). This significance remained in the adjusted model when the variables of competitive sports practice and backpack transport were added. The variables of competitive sports practice and postural habits were not associated with scoliosis in the conditional logistic regression analysis (Table 2).

**Table 2:** Association between “Scoliosis” and the selected variables, Santa Cruz, RN

	Scoliosis		Not adjusted		Adjusted	
	Absent	Present	***p***-value	OR (95%CI)	***p***-value	OR (95%CI)
	n (%)	n (%)				
**Physical activity**						
Active	31 (59.6)	21 (40.4)		1		1
Irregularly active	25 (37.3)	42 (62.7)	0.018	2.64 (1.18–5.89)	0.016	2.81 (1.21–6.49)
Sedentary	22 (59.5)	15 (40.5)	0.893	0.94 (0.37–2.38)	0.921	1.05 (0.38–2.90)
**Competitive sports practice**						
No	14 (41.2)	20 (58.8)		1		1
Yes	64 (52.5)	58 (47.5)	0.21	1.75 (0.73–4.17)	0.321	1.64 (0.62–4.37)
**Spinal posture while sitting**						
Adequate	17 (54.8)	14 (45.2)		1	–	–
Inadequate	61 (48.8)	64 (51.2)	0.565	1.25 (0.58–2.67)	–	–
**Buttocks positioning while sitting**						
Adequate	38 (50)	38 (50)		1	–	–
Inadequate	40 (50)	40 (50)	1	1 (0.52–1.92)	–	–
**Feet positioning while sitting**						
Adequate	36 (55.4)	29 (44.6)		1	–	–
Inadequate	42 (46.2)	49 (53.8)	0.299	1.37 (0.76–2.47)	–	–
**Backpack transport**						
Adequate	52 (46.2)	59 (53.2)		1		1
Inadequate	26 (57.8)	19 (42.2)	0.167	0.56 (0.25–1.27)	0.11	0.48 (0.20–1.18)
**Posture when picking up an object from the floor**						
Adequate	22 (46.8)	25 (53.2)		1	–	–
Inadequate	56 (51.4)	53 (48.6)	0.640	0.86 (0.47–1.60)	–	–
**Sleeping**						
Hammock	4 (40.0)	6 (60.0)		1	–	–
Both	24 (53.3)	21 (46.7)	0.483	0.62 (0.17–2.33)	–	–
Bed	50 (49.5)	51 (50.5)	0.583	0.70 (0.19–2.53)	–	–

When observing the practical variable description of physical activity distributed in five categories (very active, active, irregularly active A, irregularly active B and sedentary), it is noticed that there is a trend in the formation of a gradient, indicating that the lower the physical activity the student does it, the higher the prevalence of scoliosis; however, the gradient does not remain when showing those who do not practice any type of physical activity, being considered sedentary (Table 3).

**Table 3:** Descriptive analysis of physical activity and the distribution of scoliosis cases, Santa Cruz, RN, 2015

	Scoliosis	
	Absent	Present
**Physical activity**	**n (%)**	**n (%)**
Very active	9 (64.3)	5 (35.7)
Active	22 (55.0)	18 (45.0)
Irregularly Active A	15 (37.5)	25 (62.5)
Irregularly Active B	10 (40.0)	15 (60.0)
Sedentary	22 (59.5)	15 (45.5)

## Discussion

This study aimed to identify risk factors associated with scoliosis in schoolchildren by a retrospective analysis. Low physical activity and irregularly active individuals showed association with scoliosis in schoolchildren. Other variables such as postural habits and anthropometric data were not statistically significant.

Scoliosis prevalence has been reported differently in the literature. Baroni et al. (2015) [17] found 58.1% prevalence, Bueno and Rech (2015) [3] found 33.2%, whereas Hengwei et al. (2016) [24] reported 5.14%. The main factor responsible for such differences is the diagnosis method and the study setting. Although this study did not aim to estimate the prevalence of scoliosis in schoolchildren, it is worth mentioning that the Adams test was used as a diagnostic method for scoliosis.

From our sample, 23.7% did not practice physical activity, thus corroborating the findings by Bergmann et al. (2013) [25], that reported sedentary behavior prevalence of 26.8%. On the other hand, Santo, Guimarães and Galera (2011) [26] observed a prevalence of 5.2% in students who did not practice physical activity in their study. This difference may be explained because this study was carried out in a city where physical activity practice is mandatory in schools, however, despite these results, it is worth noting that our study is retrospective, different from the others which are cross-sectional studies.

The highest prevalence of irregularly active students in this study was observed in the scoliosis group. However, this association was not observed among sedentary people, diverging from the findings of Santo, Guimaraes and Galera [26], that reported a different scenario between sedentary and physical activity practitioners. This finding can be justified by the way the variable was analyzed, since the physical activity variable was distributed in several categories in this study, whereas in the study by Santo, Guimaraes and Galera [26] it was analyzed in a dichotomous way.

In a review study conducted by Mordecai and Dabke (2012) [27], the effectiveness of physical exercise for treating scoliosis was observed, and most studies showed improvement or stabilization of the scoliosis degree. The exercises ranged from trunk mobility techniques to more specific exercises such as Schroth exercises - postural exercises to correct scoliosis based on corrective postural patterns. Thus, it may be indicative that the active students, in this study, could benefit from postural and specific muscle recruitment, performed correctly, as they have higher levels of physical activity, which may differ from students with lower physical activity levels.

According to McMaster et al. (2015) [28], schoolchildren with scoliosis may feel less confident in performing physical exercises and, therefore, end up not participating in these activities when compared to their peers, which may explain the high number of sedentary people who had scoliosis observed in that study. Probably, individuals with scoliosis are afraid to worsen their condition by practicing physical activity. However, the presence of scoliosis itself should not be a limiting factor for exercise [28], as people with this condition may benefit from these activities, which involve neuromuscular feedback mechanisms in all joints [28].

In this study, 71.2% of students carried out adequate backpack transportation, and this variable was not statistically associated with scoliosis, thus corroborating the findings by Bueno and Rech (2014) [3], in which 80.7% of students carried their school material properly, but without statistical association with postural deviations. Although there is no statistical association between inadequate backpack transport and scoliosis, unilateral backpack transport can lead to overload in the spine and consequently cause back pain [29, 30]. According to Grimmer et al. (2002) [31], backpack transport with two straps presents different postural responses depending on the positioning height, as the lowest levels are more recommended for less postural responses, because it is closer to the center of gravity with lower load. On the other hand, a recent systematic review investigated the association between backpack use and low back pain and showed no association between the variables, and that may demystify aspects that the type of school material, weight and transport mode do not influence back problems [32].

When analyzing classic postural habits such as backpack transport, sitting posture, picking up an object from the floor and sleeping, no association with scoliosis was shown in the present study, despite the fact that these are predisposing factors to postural changes in clinical practice. It may be justified by the interaction of factors such as static posture and physical conditioning, in which, when asked to maintain a posture for a long period, the student can switch from a muscle relaxation with tone maintenance status to an excessive tension and joints overload status [32]. In addition, no studies that associate postural habits with scoliosis have been found, nor do postural habits appear to be associated with other spinal problems, such as low back and cervical pain [33, 34].

Complex diseases, like scoliosis, are caused by a combination of factors such as genetics, lifestyle, environment and biological conditions [35]. Thus, further studies are needed to analyze the interaction between genetic factors and behavioral and lifestyle conditions for better understanding scoliosis causal factor. In addition, further studies are needed in order to analyze postural habits such as backpack transport, backpack weight, lifestyle habits and genetic factors, observing the association with scoliosis progression and not just causal factors of the pathology, considering that scoliosis can be a progressive disease.

As limitations of this study, the restriction of cause-effect analysis can be cited because it is a retrospective study. In addition, there was informant bias, in which the student might not have discernment for the assessment instruments used; and memory bias, that is why it was necessary to adapt some questionnaires to the retrospective study design. Another limitation is related to identifying scoliosis based on the Adams test, because despite having high sensitivity and specificity, it is not the gold standard method for diagnosing scoliosis.

## Conclusion

The study showed low physical activity as a risk factor for scoliosis in schoolchildren. On the other hand, postural habits such as backpack transport, sitting posture, picking up objects from the floor and sleeping do not seem to be associated with this condition.

## Data Availability

Data and materials are available for consultation, if necessary.

## Abbreviations

CEP/HUOL: Research Ethics Committee of the Onofre Lopes University Hospital of the Federal University of Rio Grande do Norte
CI: Confidence interval
IPAQ: Daily physical activity index
OR: Odds ratios
